# Coronal Holes and Open Magnetic Flux over Cycles 23 and 24

**DOI:** 10.1007/s11207-016-1041-8

**Published:** 2016-12-27

**Authors:** Chris Lowder, Jiong Qiu, Robert Leamon

**Affiliations:** 1 Department of Mathematical Sciences, Durham University, Durham, DH1 3LE UK; 2 Department of Physics, Montana State University, Bozeman, MT 59717 USA; 3 Department of Astronomy, University of Maryland, College Park, MD 20742 USA

**Keywords:** Coronal holes, Magnetic fields, corona, Magnetic fields, models, Solar cycle, models, Solar cycle, observations

## Abstract

As the observational signature of the footprints of solar magnetic field lines open into the heliosphere, coronal holes provide a critical measure of the structure and evolution of these lines. Using a combination of *Solar and Heliospheric Observatory/Extreme ultraviolet Imaging Telescope* (SOHO/EIT), *Solar Dynamics Observatory/Atmospheric Imaging Assembly* (SDO/AIA), and *Solar Terrestrial Relations Observatory/Extreme Ultraviolet Imager* (STEREO/EUVI A/B) extreme ultraviolet (EUV) observations spanning 1996 – 2015 (nearly two solar cycles), coronal holes are automatically detected and characterized. Coronal hole area distributions show distinct behavior in latitude, defining the domain of polar and low-latitude coronal holes. The northern and southern polar regions show a clear asymmetry, with a lag between hemispheres in the appearance and disappearance of polar coronal holes.

## Introduction

Coronal holes are the observational signatures of regions of open solar magnetic field. Early observations were conducted in extreme ultraviolet (EUV) wavelengths (Tousey, Sandlin, and Purcell, 1968). Magnetic field lines within the solar corona can either connect back down to the solar surface or extend outward into the heliosphere, denoting closed and open field, respectively. In regions of open magnetic field, coronal plasma is free to follow these field lines outward, creating the solar wind and a plasma density depletion at the field footpoints. Appearing as dark regions in X-ray and EUV images, coronal holes correspond with reduced emission from solar plasma localized to open magnetic field footpoints (Wang, 2009). Cranmer (2009) discusses a complete review of coronal holes in exhaustive detail.

The Sun’s open magnetic field and coronal hole structure varies alongside the overall solar activity cycle, running through a magnetic polarity swap every 11 years. The connection between coronal hole activity and open magnetic flux throughout the solar cycle has been considered by several studies (Harvey and Recely, 2002; Wang, 2009). Harvey and Recely (2002) assessed the properties of polar coronal holes throughout Solar Cycles 22 and 23. With the help of a series of He i 10,830 Å spectroheliograms, polar coronal holes were identified over the period 1989 September to 2002 March, and their area and the enclosed magnetic flux was measured. The authors found that polar coronal holes throughout this period initially develop at lower latitudes of about 50 – 60 degrees, and extend to the poles within three subsequent rotations. These polar coronal holes then have an observed lifetime of between 8.3 and 8.7 years. An asymmetry was observed in the timing of the initial appearance and evolution of coronal holes at each pole, with a difference of several months between poles. This mirrors earlier observations by Webb, Davis, and McIntosh (1984), who found a lag of 9 and 6 months between polar coronal hole appearances between Cycles 19 – 20 and 20 – 21, respectively. The relative areal extent of each polar coronal hole can also show an asymmetry from one solar cycle to the next (Broussard *et al.*, 1978; Sheeley, 1980). Wang and Sheeley (2002) explored in detail the relationship between active region evolution and consequent coronal hole distribution through observation and modeling. In particular, they noted the evolution of polar coronal holes as a consequence of remaining flux that is transported from lower latitudes. More recent work by McIntosh *et al.* (2013) explored the hemispheric asymmetry of photospheric magnetism throughout Cycle 23 and the early stages of Cycle 24. While He i 10,830 Å observations of coronal holes can provide accurate results in polar regions, there is discrepancy at lower latitudes (Kahler, Davis, and Harvey, 1983; Schrijver and De Rosa, 2003; Malanushenko and Jones, 2005).

Despite the relatively long history of observations of the solar activity cycle, continuous and full-disk magnetic field and solar corona observations extend back only to cover the previous two cycles. The *Solar and Heliospheric Observatory* (SOHO) began this observational campaign from 1995 through 2011, with results from the *Solar Dynamics Observatory* (SDO) picking up since May 2010. McIntosh *et al.* (2014) recently conducted a comprehensive analysis of observations by these spacecraft, showing that distributions of EUV bright points and structures of surface magnetic elements, characterized by the magnetic range of influence (MRoI), both migrate from high latitudes (${\pm}\,55~\mbox{degrees}$) toward the equator in a time period of 19 years. In addition, the evolution of low-latitude coronal holes and records of coronal green line emissions also overlaps with this trend. Robbrecht *et al.* (2010) explored this apparent extended cycle as observed from coronal emission in simulations of green line emission and *Extreme ultraviolet Imaging Telescope* (EIT) EUV observations. They suggested that these overlapping band structures, rather than indicating an extended cycle, are merely remnants of reconnection between low latitude and polar flux from the current cycle. Observing the progress of the activity bands formed by these very different features helps decipher the underlying solar magnetism in a cycle structure, and can help to shed additional light on these processes.

Among these features, coronal holes map the footprints of open magnetic field at locations that vary with the solar cycle. McIntosh *et al.* (2014) showed that low-latitude coronal holes migrate toward the equator as the cycle progresses, whereas during solar minimum, holes exist primarily in polar regions, where EUV bright points and coronal green line emission is sparse. These areas are of crucial importance in the solar magnetic activity cycle. The polar region is usually dominated by a magnetic field of one polarity, and each cycle begins with the reversal of this polarity at the end of the solar minimum, when the meridional convective flow has recycled the field to the poles and flux cancellation takes place (Upton and Hathaway, 2014). Existing models of the global solar magnetic field often make the point to focus on a comparison of computed open field models with the observed coronal holes at the poles, or with the total heliospheric open magnetic flux (Mackay, Priest, and Lockwood, 2002a,b; Yeates *et al.*, 2010). On the other hand, much effort is also needed to examine coronal holes evolving across the latitudes. Tracking open magnetic flux on the Sun following coronal hole evolution from the poles to the low latitudes adds a valuable piece to the puzzle of the solar magnetic activity cycle.

The continuous full-disk coronal and magnetic field observations in the past two decades provide the opportunity to study the evolution of coronal holes and open magnetic flux in Solar Cycles 23 and 24. Lowder *et al.* (2014) developed an automated technique to detect persistent coronal holes and characterize their properties using a database of full-disk EUV images obtained by several spacecraft instruments over a time span from 1996 May to 2013 January. In this present study, we use the same technique and the same database, which is further extended to August 2014, however. The extended database covers a significant portion of the current Solar Cycle 24, and hence provides an opportunity to compare coronal hole properties between the past Cycle 23 and this special new cycle, which, after an unexpectedly deep minimum, slowly started around 2010, and rose to a low maximum in early 2014 (McIntosh *et al.*, 2015).

Lowder *et al.* (2014) have also measured coronal hole areas, and the unsigned and signed total flux measured in these holes, distinguishing polar regions from low latitudes with an arbitrary division at ${\pm}\,65~\mbox{degrees}$. This article presents a more careful and detailed examination of the latitude dependence of coronal hole properties, where a contrast between the consecutive two cycles is apparent. A brief review of the database and technique is presented in Section 2. Section 3 presents the latitude dependence of coronal hole properties and their variations from the last cycle to the current cycle, followed by conclusions and discussions in Section 4.

## Method and Data

EIT (Delaboudinière *et al.*, 1995), an instrument onboard SOHO, has provided 14 years of nearly continuous EUV observations over the span of Solar Cycle 23. SDO was launched in 2010, continuing the role of a provider of synoptic EUV observations through the EUV telescope *Atmospheric Imaging Assembly* (AIA, Lemen *et al.*, 2012). Having launched a few years before, the twin spacecraft *Solar Terrestrial Relations Observatory* (STEREO) A and B have swept out a significant portion of vantage points on the far side of the Sun. Employing the *Extreme Ultraviolet Imager* (EUVI, Howard *et al.*, 2008) on each of the STEREO spacecraft, additional viewpoints are available in the EUV. The particular combination of EUV data from SDO/AIA and STEREO/EUVI A/B allows for a unique observational opportunity. As the STEREO spacecraft have swept out in their orbits, expanded heliographic longitude coverage in EUV has been made possible. Additionally, polar EUV coverage was expanded by the more staggered $B$-angle for each of the spacecraft in use. Joint observations by the three spacecraft complement one another to provide better coverage of the polar areas, important regions contributing to the evolution of solar open magnetic flux. The joint AIA/EUVI coverage makes possible continuous, consistent, and nearly full solar surface observations of coronal hole boundaries over Solar Cycle 24. Table 1 displays the relevant datasets used in this study and the corresponding availability ranges. Using these data ranges, we characterize coronal hole boundaries over one and a half solar activity cycles, and compare the results between Cycles 23 and 24.

**Table 1:** Data coverage for each instrument source.

Source	Observable	Start		End	
		CR	Date	CR	Date
SOHO/EIT	EUV 195 Å	1909.96	1996 05 31	2105.27	2010 12 31
SDO/AIA	EUV 193 Å	2096.76	2010 05 13	2153.92	2014 08 19
STEREO/EUVI	EUV 195 Å	2096.76	2010 05 13	2153.92	2014 08 19
WSO Harmonics	$B_{r}$	1893.00	1995 02 23	2140.00	2013 08 04
Synoptic SOHO/MDI	$B_{r}$	1911.00	1996 06 28	2104.00	2010 11 26
Synoptic SDO/HMI	$B_{r}$	2096.00	2010 04 22	2156.00	2014 10 14

**Figure 1:**
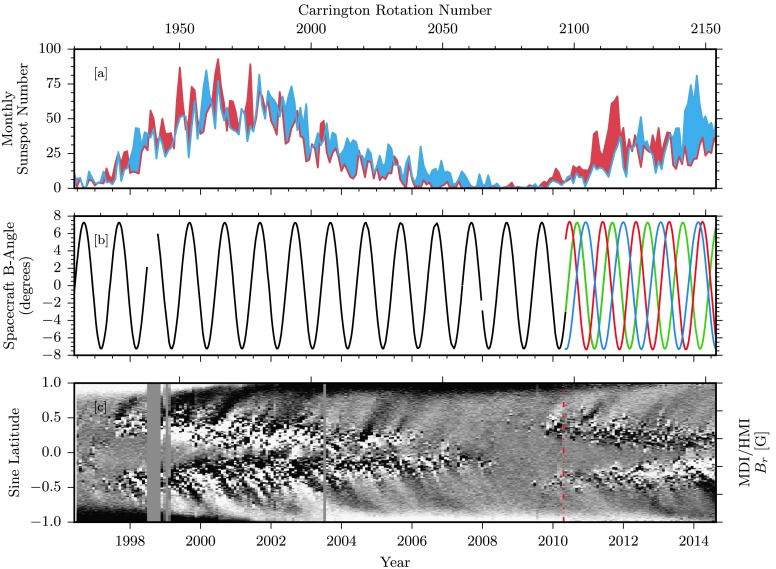
[a] Hemispheric sunspot number for northern and southern hemispheres in red and blue, respectively. (WDC-SILSO, Royal Observatory of Belgium, Brussels.) [b] Spacecraft solar $B$-angle over the course of observations in Table 1. SOHO/EIT (black), SDO/AIA (green), STEREO/EUVI A (red), and STEREO/EUVI B (blue) angles are displayed. [c] Butterfly diagram (latitude-time profile) of the mean radial magnetic field strength, scaled between ${\pm}\,5~\mbox{G}$. Measurements from SOHO/MDI transition to those from SDO/HMI at the vertical dot-dashed red line in early 2010.

To provide context for the solar cycle dependence of coronal hole properties, Figure 1 displays the observational spacecraft $B$-angle in comparison with the position in the solar activity cycle. Part of this dataset was featured in our previous study. However, here the range is extended to cover through August 2014 (Lowder *et al.*, 2014, Figure 13). The upper panel displays the monthly hemispheric sunspot number (WDC-SILSO, Royal Observatory of Belgium, Brussels), with the northern and southern sunspot counts in red and blue, respectively, and with the dominant pole shade filling between. The middle panel displays the spacecraft $B$-angle for SOHO/EIT (black), SDO/AIA (green), STEREO/EUVI A (red), and STEREO/EUVI B (blue). Using the single vantage point of SOHO/EIT, our polar observations are limited by this angle. This will become apparent in subsequent figures. Rather than attempting a convoluted method to correct for or estimate this lack of coverage, results are displayed unmodified. With the combination of SDO/AIA and STEREO/EUVI A/B, this polar coverage gap is greatly reduced. When we compare this with our gauge of the solar activity cycle, we note that our EIT observations span over the entire first solar cycle under consideration. Our improved polar coverage from STEREO begins conveniently just as the next solar cycle begins. The bottom panel displays a butterfly diagram of the mean radial magnetic field strength, taken from measurements from the *Michelson Doppler Imager* (MDI, Scherrer *et al.*, 1995) and the *Helioseismic and Magnetic Imager* (HMI, Scherrer *et al.*, 2012), with a vertical dot-dashed red line to indicate the transition. This dataset is scaled to ${\pm}\,5~\mbox{G}$ to highlight trends in dominant magnetic field sign throughout the solar cycle.

With these data, techniques have been developed and documented for the automated detection of coronal hole boundaries, detailed in the literature (Lowder *et al.*, 2014). In brief, each frame of EUV data is analyzed via an instrument-independent intensity thresholding method to mark out coronal hole boundaries. Boolean maps of suspected coronal hole regions are generated and mapped into a Carrington Equal Area (CEA) projection, and then segmented and cataloged into individual regions through a watershed method. For our SOHO-era (solar activity Cycle 23) data, this process is repeated for each time-step, with half of the solar surface not visible. Full rotation maps are built by summing these individual EIT hole maps over each Carrington rotation, and then flattening into a boolean map. For the SDO-era (solar activity Cycle 24) data, thresholded projections are made individually for each of our three datasets (SDO/AIA and STEREO/EUVI A/B), and then summed and flattened into one resulting boolean map.

This initial mapping detects both coronal holes and filament channels, which both appear as dark features in EUV. Here we use the underlying magnetic flux density information to distinguish the two. Filament channels straddle magnetic polarity inversion lines, encompassing roughly equal distributions of positive and negative flux. In the encompassed area of a coronal hole, a single magnetic polarity dominates (Cranmer, 2009). Therefore, we calculate the skew of magnetic flux density in each candidate coronal hole region, using magnetograms from SOHO/MDI in conjunction with SOHO/EIT, or SDO/HMI with SDO/AIA and STEREO/EUVI A/B. Regions with a relatively small skew are considered to be filament channels and are therefore ‘sieved’ out, leaving behind a boolean CEA map of coronal hole pixel locations.

For the current application of this routine for long-duration coronal hole evolution, the EUV data cadence is as follows. SOHO/EIT data are assimilated daily, with a resulting daily coronal hole map. These daily maps are then summed and flattened to provide an upper estimate over an entire solar rotation, to account for missing far-side data. SDO/AIA and STEREO/EUVI A/B data are gathered at a cadence of 12 hours, providing two full-surface coronal hole maps per day. Note that with the expanded longitudinal and polar coverage, these maps are considered individually, without the requirement of summing over an entire solar rotation.

For more details and intermediate results on this process, see Lowder *et al.* (2014). For the sake of brevity, this code and existing methodology are referred to as the Global Automated Coronal Hole Detection routine (GACHD). Here the techniques have been further refined, and applied to the complete data range from 31 May 1996 to 19 August 2014, the furthest data available until the recent communication loss with one of the STEREO spacecraft. These data cover the entire past Cycle 23 as well as a large portion of the current Solar Cycle 24.

## Coronal Hole Properties in Solar Cycles 23 and 24

Using this dataset of coronal hole boundaries, spanning over one and a half solar cycles in length, we can consider several interesting quantities. These include the area as well as the signed and unsigned magnetic flux enclosed by coronal hole boundaries. Rather than considering just the polar coronal hole properties, our data extend across all latitudes to characterize the latitude-dependence of these properties and their evolution.

**Figure 2:**
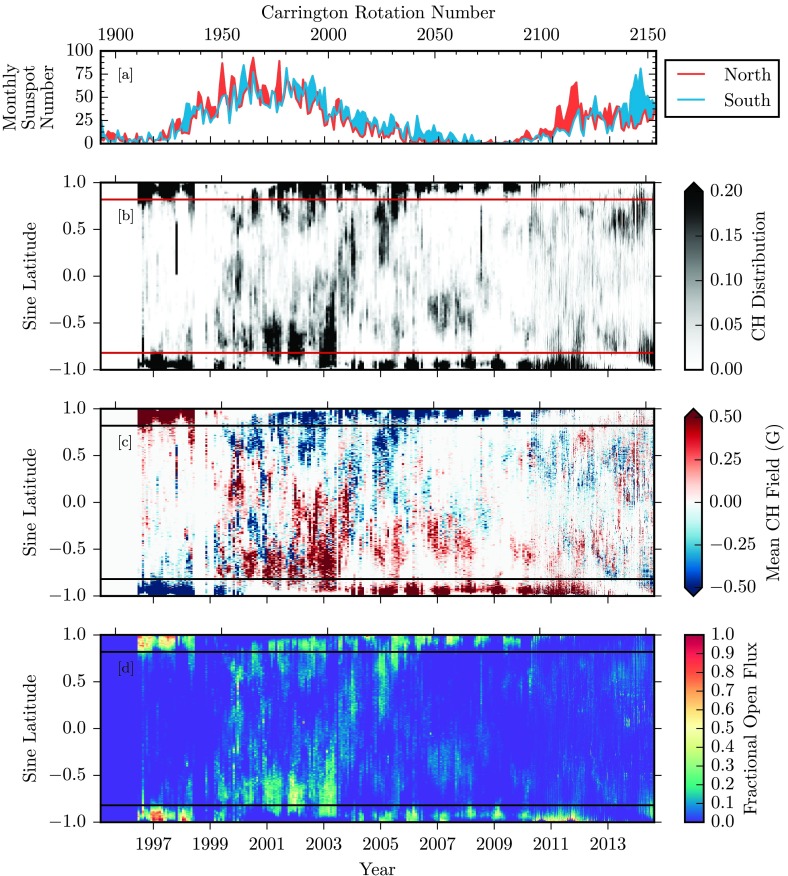
For the time period 31 May 1996 to 19 August 2014, we show [a] the monthly hemispheric sunspot number for the northern (red) and southern (blue) hemispheres (WDC-SILSO, Royal Observatory of Belgium, Brussels). [b] The coronal hole latitude profile of the distribution, integrated over longitude. Horizontal red lines are marked at ${\pm}\,55~\mbox{degrees}$ latitude to distinguish polar and low-latitude zones. [c] The latitude profile of the coronal hole dominant polarity, integrated over longitudes. [d] The latitude profile of coronal hole-enclosed magnetic flux, integrated over longitudes.

**Figure 3:**
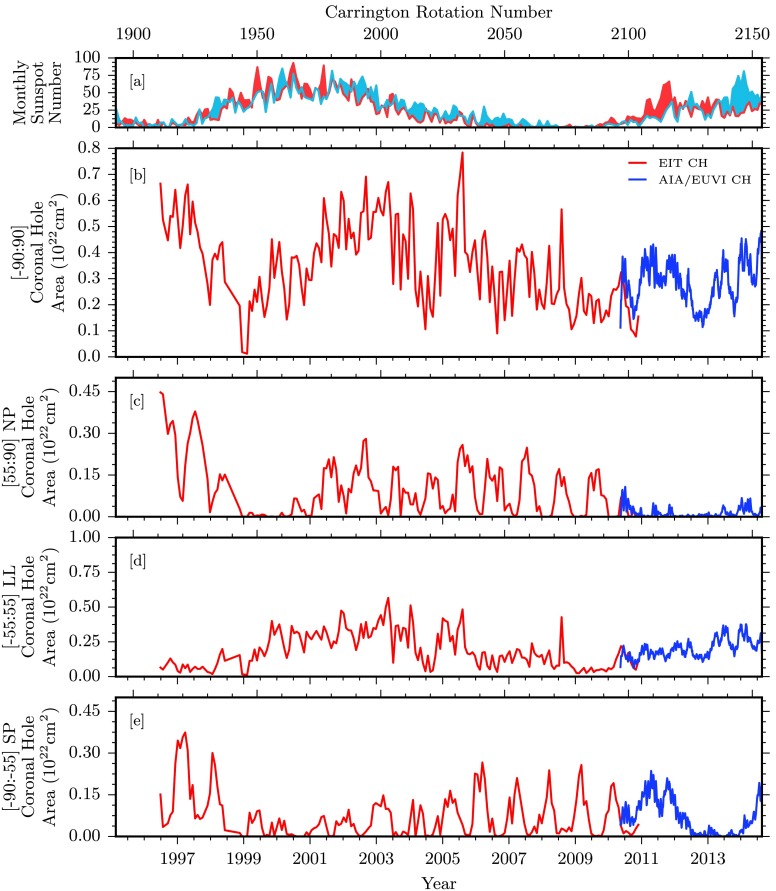
Comparisons of coronal hole area and associated quantities over the entire data span. [a] Hemispheric sunspot number for northern and southern hemispheres in red and blue, respectively (WDC-SILSO, Royal Observatory of Belgium, Brussels). [b] Total coronal hole area for some of our data sources. EIT and AIA/EUVI coronal hole boundaries are used to compute the coronal hole area shown in red and blue, respectively. [c – e] Coronal hole area computed for northern polar, low latitudinal, and southern polar regions.

**Figure 4:**
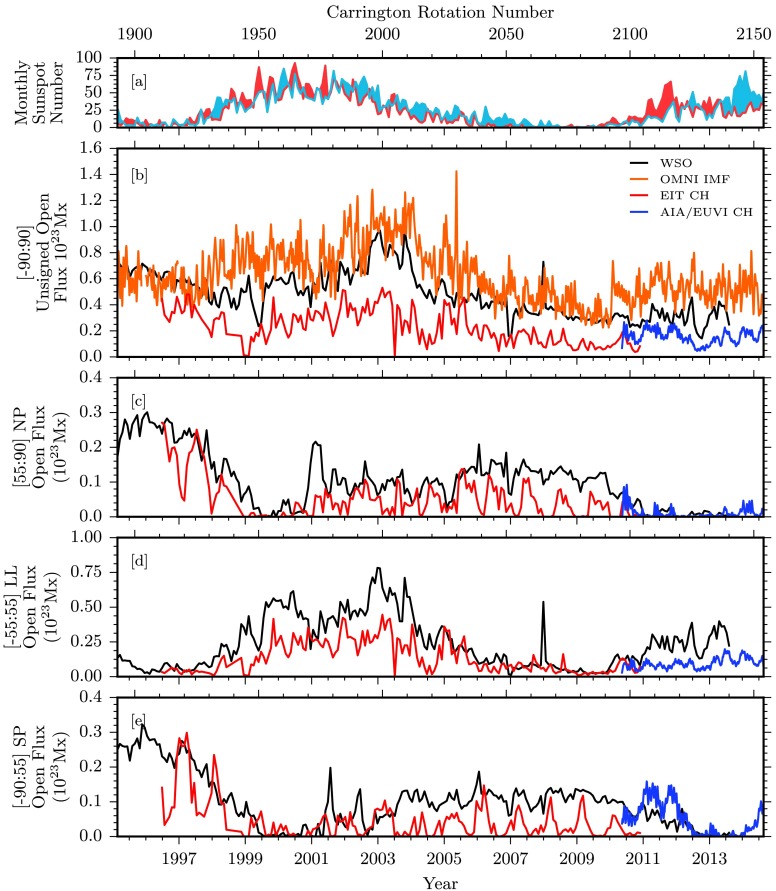
Comparisons of unsigned photospheric flux and associated quantities over the entire data span. [a] Hemispheric sunspot number for northern and southern hemispheres in red and blue, respectively (WDC-SILSO, Royal Observatory of Belgium, Brussels). [b] Total unsigned open magnetic flux for some of our data sources. WSO data in black are computed from the total open field boundaries. EIT and AIA/EUVI coronal hole boundaries are used to compute the total enclosed magnetic flux in red and blue, respectively. OMNI $|B_{r}~(r=1~\mbox{AU})|$ is used to compute an equivalent open magnetic flux, displayed in orange. [c – e] Open magnetic flux computed for northern polar, low latitudinal, and southern polar regions.

**Figure 5:**
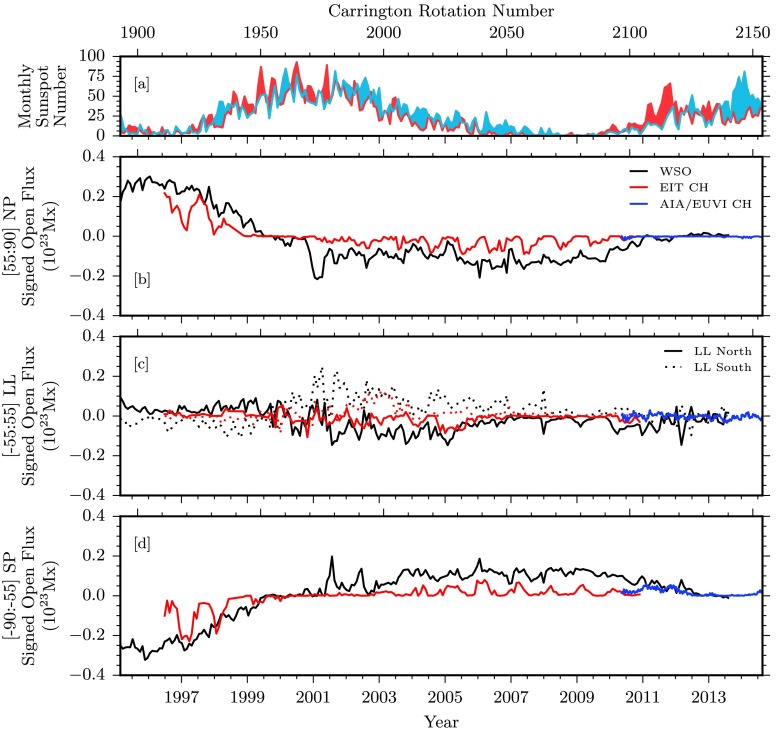
Comparisons of signed photospheric flux and associated quantities over the entire data span. [a] Hemispheric sunspot number for northern and southern hemispheres in red and blue, respectively (WDC-SILSO, Royal Observatory of Belgium, Brussels). [b – d] Signed open magnetic flux for some of our data sources for the northern polar, low-latitudinal (northern and southern portions in solid and dotted styles), and southern polar regions. WSO data in black are computed from total open field boundaries. EIT and AIA/EUVI coronal hole boundaries are used to compute the total enclosed magnetic flux shown in red and blue, respectively.

**Figure 6:**
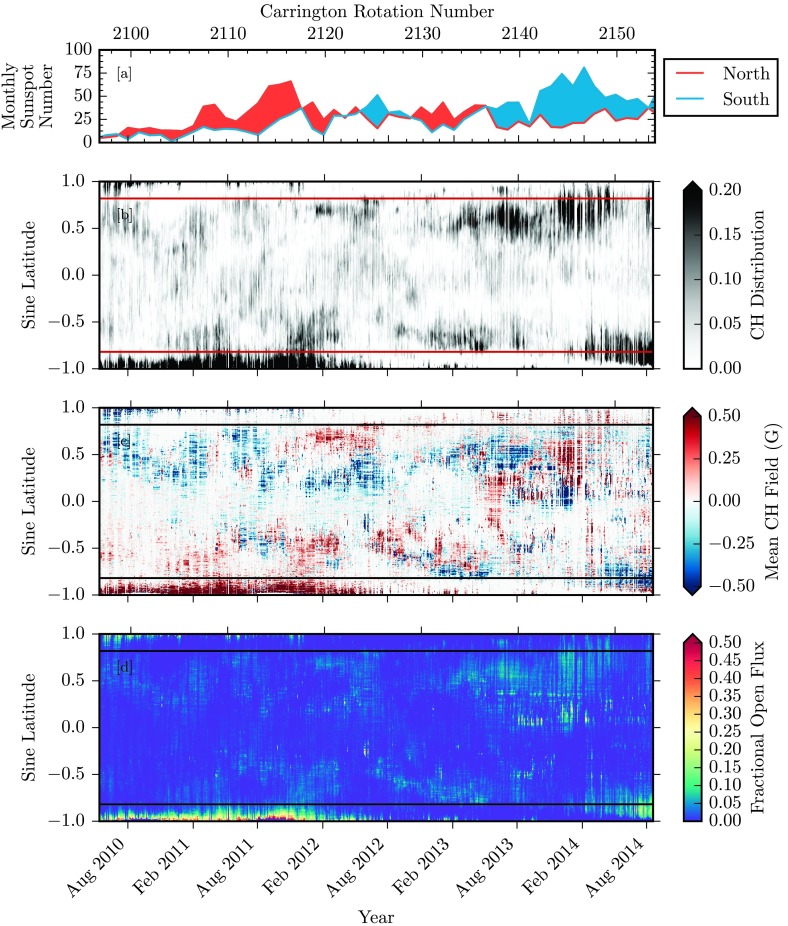
For the time period 13 May 2010 – August 19 2014 we show [a] the monthly hemispheric sunspot number for the northern (red) and southern (blue) hemispheres (WDC-SILSO, Royal Observatory of Belgium, Brussels). [b] The coronal hole latitude profile distribution, integrated over longitude. Horizontal red lines are marked at ${\pm}\,55~\mbox{degree}$ latitude, to distinguish polar and low-latitude zones. [c] The latitude profile of the coronal hole dominant polarity, integrated over longitudes. [d] The latitude profile of the coronal hole enclosed magnetic flux, integrated over longitudes.

**Figure 7:**
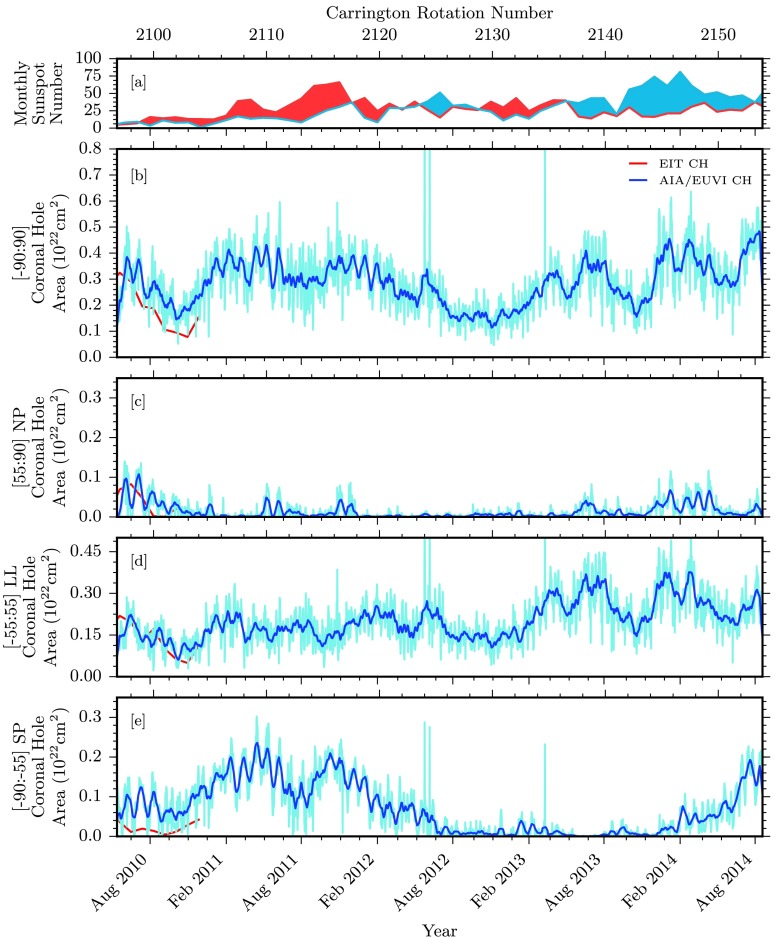
Comparisons of coronal hole area and associated quantities over the SDO-era data span. [a] Hemispheric sunspot number for northern and southern hemispheres in red and blue, respectively (WDC-SILSO, Royal Observatory of Belgium, Brussels). [b] Total coronal hole area for some of our data sources. EIT and AIA/EUVI coronal hole boundaries are used to compute the coronal hole area in red and blue, respectively. AIA/EUVI raw data are displayed in light blue, with a 27-day running average displayed in dark blue. [c – e] Coronal hole area computed for northern polar, low latitudinal, and southern polar regions.

**Figure 8:**
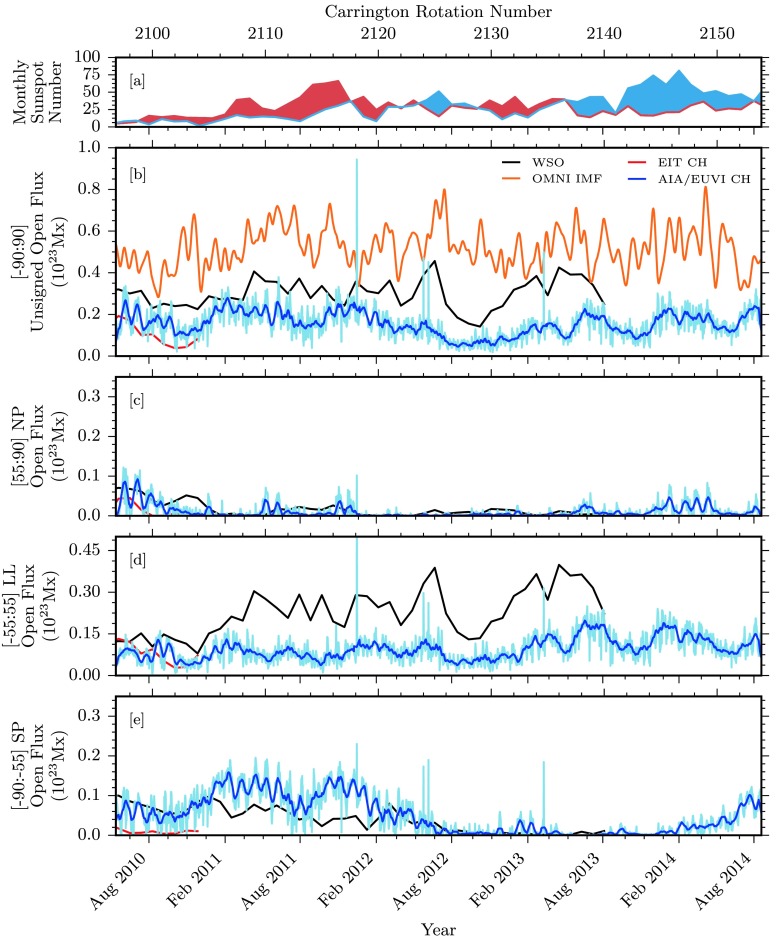
Unsigned photospheric flux and associated quantities over the SDO-era data span. [a] Hemispheric sunspot number for the northern and southern hemispheres in red and blue, respectively (WDC-SILSO, Royal Observatory of Belgium, Brussels). [b] Total unsigned open magnetic flux. The WSO-computed open flux is displayed in black. EIT and AIA/EUVI coronal hole boundaries are used to compute the total enclosed magnetic flux in red and blue, respectively. AIA/EUVI raw data are displayed in light blue, with a 27-day running average displayed in dark blue. OMNI $|B_{r}~(r=1~\mbox{AU})|$ is used to compute an equivalent open magnetic flux (orange). [c – e] Open magnetic flux computed for northern polar, low latitudinal, and southern polar regions.

**Figure 9:**
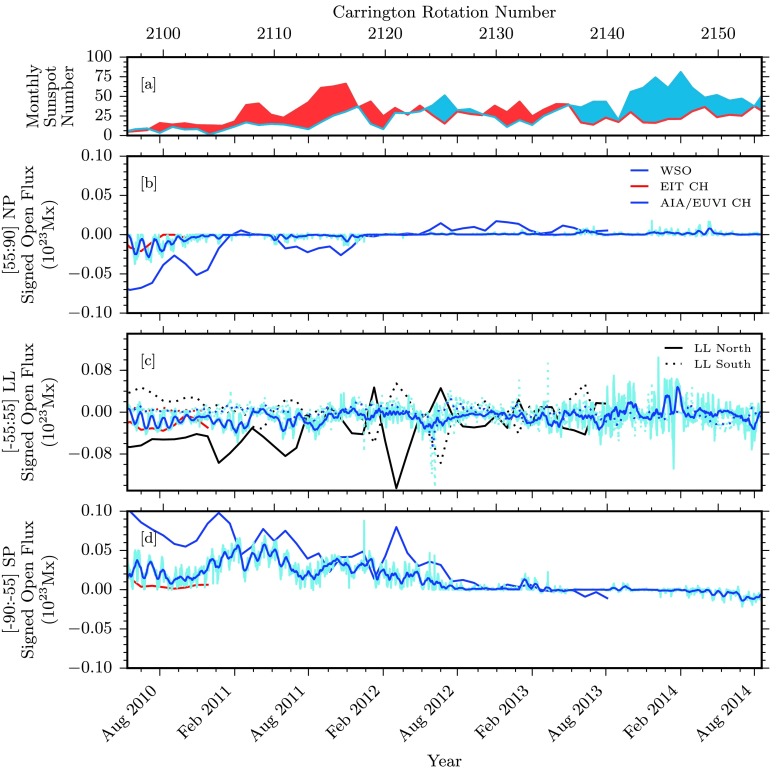
Comparisons of signed photospheric flux and associated quantities over the SDO-era data span. [a] Hemispheric sunspot number for northern and southern hemispheres in red and blue, respectively (WDC-SILSO, Royal Observatory of Belgium, Brussels). [b – d] Signed open magnetic flux for some of our data sources for the northern polar, low-latitudinal (northern and southern portions in solid and dotted styles), and southern polar regions. The WSO data in black are computed from total open field boundaries. EIT and AIA/EUVI coronal hole boundaries are used to compute the total enclosed magnetic flux in red and blue, respectively. AIA/EUVI raw data are displayed in light blue, with a 27-day running average displayed in dark blue.

In this section, we first present the time-latitude maps of coronal hole properties. The total magnetic flux in the holes is then presented by comparing the polar regions with low latitudes, and the past Cycle 23 with the current cycle. For the following set of figures, there are two notes of importance. Figures 2, 3, 4, and 5 display quantities over the full time span from May 1996 to August 2014, illustrating the solar cycle dependence of the measured properties. Figures 6, 7, 8, and 9 provide a zoomed-in display of identical quantities from 2010 May onward, over the SDO and STEREO-era data for Solar Cycle 24. Over all of these plots, panel a displays the monthly hemispheric sunspot number in red and blue for the northern and southern sunspot counts, respectively. This panel will lead some of the following figures for an easily markable solar activity cycle cross-reference tool.

It should be noted that a partial overlap with the results in Lowder *et al.* (2014) exists within Figures 3, 4, and 5. However, results contained here use an alternative latitudinal definition of polar regions. Additional data extend the range of results to cover a more significant fraction of Cycle 24.

### Latitude Coronal Hole Profiles

We consider Figure 2, which displays the full latitude dependence of our coronal hole quantities over the full time span of our datasets. Of immediate note, the distributions of the coronal hole area (panel b), the mean magnetic field (panel c), and the total unsigned magnetic flux (panel d) in the holes all exhibit distinctive evolution patterns near ${\pm}\,55~\mbox{degrees}$ latitude, which are marked by two horizontal lines in the figures.

Other authors have arrived at slightly different limiting latitudes for polar coronal hole boundaries. Wang and Sheeley (2002) considered the formation and evolution of a polar field as beginning at far lower latitudes. In this formalism, remnants of active-region flux are transported toward the poles, where shearing and diffusion work to reduce the nonaxisymmetric component of this field. The remaining field component continues toward the poles, establishing a dominant polarity. In this manner, coronal holes begin their formation in lower latitudes, ${\pm}\,30\,\mbox{--}\,60~\mbox{degrees}$, before establishing themselves above ${\pm}\,60~\mbox{degrees}$, with boundaries somewhat maintained by a balance between meridional flow and diffusion.

Similarly, from observations of polar coronal holes in He i 10,830 Å spectroheliograms, Harvey and Recely (2002) found that most held positions above ${\pm}\,60~\mbox{degrees}$, with some exceptions. Taking the mean polar coronal hole latitude, Harvey and Recely (2002) found dips in the northern and southern poles to 58 – 59 degrees and 54 – 55 degrees, respectively, during the minimum between Cycles 22 and 23. In the following analysis, the ${\pm}\,55~\mbox{degree}$ latitude is therefore chosen as the division between polar and low-latitude regions. While lower than the ${\pm}\,60~\mbox{degree}$ boundary, this provides a clear distinction of polar regions, without misattributing open magnetic flux. Note that this distinction of polar regions differs from the previous study by Lowder *et al.* (2014), which used the ${\pm}\,65~\mbox{degree}$ latitude as a separator. Appendix A works through a more detailed analysis of the effects of shifting this polar latitude boundary, with further justification for this boundary choice. Note that the selection here of a polar latitude is useful for comparative purposes.

These particular latitudes at ${\pm}\,55$-degrees have been noted previously as a demarcation of certain other aspects of solar activity, coincidentally. Thompson *et al.* (1996), using the *Global Oscillation Network Group* (GONG) set of observations, considered the internal rotation rate of the Sun from the surface deep into the convection zone. A slice through the convection zone reveals the internal structure of the differential rotation, beneath the photospheric surface flow. At ${\pm}\,55~\mbox{degree}$ latitude, there is a distinct change in the behavior of these subsurface flows. Spruit (2011), in an effort to sketch out a few theories of internal solar dynamo processes, devised a simple model. Starting with a uniform poloidal field, a differential rotation profile from observations was applied. This resulted in a shearing of the field, which can eventually lead to a buildup of an azimuthal field and an instability. From this simple model, the azimuthal field builds as $\partial_{t} B_{\phi} \sim\sin2 \lambda(1+ 1.51 \sin^{2} \lambda )$. As a function of latitude, the maximum shearing occurs around ${\pm}\,55~\mbox{degrees}$. Snodgrass (1983) finds this same latitude as a peak of differential rotation shearing. This latitude was further revealed as a zone of interest by McIntosh *et al.* (2014, 2015), who found a scarcity of EUV bright points above this latitude, as well as a distinct evolution pattern of the magnetic range of influence (MRoI) divided by this latitude. While the coronal hole polar boundary is determined by properties of the polar field itself, several other quantities show variation nearby.

Panel b of Figure 2 displays for each time frame and bin of sine latitude the fraction of longitude bins that are characterized as coronal holes, with the color mapping truncated at 0.2 for display purposes. Note that this fraction varies from 0 (no coronal holes at any longitudes at that particular latitude) to 1.0 (complete coronal hole coverage at every longitude for that particular latitude). From 1996 to 1998, and from 2006 through 2012, coronal holes are concentrated in the polar regions above ${\pm}\,55$-degrees. Note here that the polar hole extensions from 1996 to 1998 extend further equatorward, pushing toward our boundaries at ${\pm}\,55~\mbox{degrees}$. The apparent annual variation in the polar hole area is not a physical result, but rather a consequence of the solar $B$-angle modulation of the SOHO spacecraft (see Figure 1). The single vantage point of these observations results in alternating reduced polar coverage. From 2010, with the combination of the AIA/EUVI suite of data, staggered spacecraft $B$-angles allow for more comprehensive polar measurements. During the solar maximum from 1999 to 2003, and then from 2012 through 2014, coronal holes extend to low latitudes below ${\pm}\,55~\mbox{degrees}$, and are diminished in polar regions.

Figure 2, panel c, displays the mean magnetic field strength encompassed by coronal hole boundaries, averaged over all longitude bins for each bin of sine latitude. Using synoptic magnetograms from SOHO/MDI and SDO/HMI, coronal hole enclosed magnetic flux (signed and unsigned) is integrated over all longitudes. To avoid the contribution of noise for both signed and unsigned flux calculations, instrument-specific noise levels are used to remove magnetic flux density measurements below this noise threshold. For MDI synoptic chart measurements of radial magnetic flux density, this noise level is measured as ${\pm}\,5.0~\mbox{G}$, while for similar synoptic measurements from HMI, this noise level is measured as ${\pm}\,2.3~\mbox{G}$ (Liu *et al.*, 2012). While the range of integrated values extends from −8.4 to 14.3 G, here the map is displayed truncated at ${\pm}\,0.5~\mbox{G}$ to illustrate the frequently weak polarity dominance at lower latitudes. The resulting map displays the distribution of the dominant polarity of coronal hole-enclosed magnetic flux and its evolution over the solar cycle. These distributions of dominant coronal hole polarity exhibit a hemispheric pattern over the course of the solar cycle. In the rise of the previous cycle before 1999, the northern polar coronal hole distribution is dominated by positive magnetic field, with the southern polar coronal hole dominated by negative field. At lower latitudes, this same general polarity trend is observed, although at a much lower strength, usually below 1 G, and the trend is not as spatially uniform across latitudes and time. During the solar activity maximum in 2001 – 2003, the hemispheric field reversal is observed in the swap of the polar coronal hole dominant polarity, persisting until 2012. For the current cycle, the reversal of the dominant polarity in polar regions begins in 2012 and 2013 for the northern and southern hemisphere, respectively. However, the resulting polar coronal hole coverage and polarity dominance have been very weak in comparison with the previous cycle. In lower latitudes during the current cycle, the distribution of coronal hole polarity dominance is much weaker and spatially mixed.

Figure 2, panel d, displays the fractional unsigned open flux, collected by summing all coronal-hole-encompassed-flux over longitude bins for each time and sine-latitude bin. An identical noise cutoff of the synoptic magnetogram is used for this calculation to avoid including instrument noise. Note that the range of calculated open flux extends from $0 \mbox{ to } 4.7\times 10^{20}~\mbox{Mx}$, with the displayed data being represented as a fraction of the maximum measured. With a sine-latitude coordinate system, broken into 1440 pixel latitude bins, each pixel represents an equal solar surface area. This allows a comparison of the relative strength of open flux sources at polar and low-latitude regions. From 1997 – 1999, the polar coronal holes contain the majority of the open magnetic flux. As the poles reverse as solar activity increases, larger concentrations of open flux appear at lower latitudes between 1999 and 2003. Past the maximum of Cycle 23, this pattern quickly returns to larger concentrations of open flux in the poles, until the next reversal around 2013. In the southern pole, the concentration of coronal hole flux persists for the extended deep minimum until the next reversal around 2013. This particular cycle from 2009 onward has shown unique behavior in the early drop-off of the northern polar coronal hole signature. This in turn has resulted in almost nonexistent open flux from the northern polar region.

In concluding this portion of the analysis, we note that McIntosh *et al.* (2014, 2015) have also reported the solar-cycle dependence of coronal holes observed in much the same time range. However, their study only captured coronal holes with area greater than $20{,}000~\mbox{Mm}^{2}$ at low latitudes (below ${\pm}\,55~\mbox{degree}$). By employing joint observations by AIA/EUVI, our study not only provides a more complete coverage of polar holes from May 2010, but also allows the assessment of polar hole measurements by EIT in the past cycle through comparison of measurements by different instruments, as we discuss further in Sections 3.2 and 3.3.

### Coronal Hole Area

Figure 3 breaks down the latitude dependence of the coronal hole areas in Figure 2 into the distinct ranges of northern polar (NP; 55:90 degrees), low latitudinal (LL; −55:55 degrees), and southern polar (SP; −90:−55 degrees). Results from EIT and the combination of results from AIA/EUVI are displayed in red and blue, respectively. Note here that EIT measurements of coronal hole area are integrated over each solar rotation, providing a measurement within observable regions. The brief overlap of EIT and AIA/EUVI observations in the second half of 2010 demonstrates that as also discussed in Lowder *et al.* (2014), the joint AIA/EUVI observational coverage allows better estimates of polar coronal hole areas and fluxes.

Figure 3, panel b, displays results for the entire range of latitudes, from −90:90 degrees. The total area of coronal holes over the course of this study varies between $1\,\mbox{--}\,7\times 10^{21}~\mbox{cm}^{2}$, or from 2 % to 12 % of the total solar surface area. Figure 3, panels c – e, displays the latitude zones under consideration, where we can note several observations. The northern coronal hole has dropped from 2011 onward, hovering around zero area, and has never recovered to the pre-2009 level. The southern pole area was still rising from 2011, and suffered a drop in the middle of 2012, but recovered from 2014 onward. Compared with the previous cycle drop in polar coronal hole area from 1999 – 2001, this behavior is highly asymmetric, reflecting the asymmetries in sunspot activity that are visible in Figure 1.

In both cycles, low-latitude coronal holes cover a large area during the solar maximum, and consequently are a source of a significant amount of total open flux on the Sun during the solar maximum. In the current Cycle 24, the low-latitude coronal hole area is notably smaller than in the last cycle.

### Unsigned Magnetic Flux

In a similar manner, Figure 4 breaks down the latitude dependence of the unsigned open magnetic flux encompassed by our coronal hole boundaries. Figure 4, panel b, displays the total unsigned open flux, with several datasets in place. EIT and AIA/EUVI results for coronal hole-encompassed unsigned flux are plotted in red and blue, respectively. These direct measurements are compared with the open flux calculated by the potential field source surface (PFSS) model (Schatten, Wilcox, and Ness, 1969; Wang and Sheeley, 1992). This model calculates the magnetic potential field using spherical harmonics of photospheric radial magnetic field determinations from the *Wilcox Solar Observatory* (WSO), and with the source-surface defined at $2.5R_{\odot}$, where field lines are assumed to be open. An array of field lines is traced down from this source-surface to their photospheric origin, and is mapped out as the boundaries of the open magnetic field. Magnetic fluxes are then computed by integrating the photospheric radial magnetic field within these open footpoint boundaries. Note that while WSO observations are used as a boundary condition, the calculated open flux is a PFSS model output using this observational input. For the purposes of comparison with other model results, this dataset may be referred to as ‘observational,’ referring to the observational basis of the model input. The resulting unsigned open magnetic flux from this PFSS model is displayed in black.

The measured open flux from the EIT coronal holes undershoots the values predicted by the PFSS model, which is partially due to a contrast issue with SOHO/EIT (Lowder *et al.*, 2014). This issue is most prevalent in lower-latitude regions during periods of higher solar activity. AIA/EUVI measurements reveal more open flux than EIT alone during the overlapping period in the second half of 2010, as a result of the more complete coverage of the polar regions by the AIA/EUVI dataset. This additional flux sets the AIA/EUVI dataset more in line with the PFSS model.

Finally, the OMNI dataset is used to compute an equivalent open magnetic flux. In situ (Lagrange point L1) measurements of the $B_{x}$ component (Geocentric Solar Ecliptic; GSE) of the magnetic field extend backward for decades through a cross-spacecraft calibrated dataset (King and Papitashvili, 2005). The OMNI data are obtained from the GSFC/SPDF OMNIWeb interface at http://omniweb.gsfc.nasa.gov. For this study we focus on data for the previous two decades. When we assume that our long-term interplanetary magnetic field is uniform and radial, the equivalent OMNI unsigned open magnetic flux can be computed as $\Phi_{\mathrm{OMNI}} = 4 \pi R_{L1}^{2} |B_{x}|$ (Lockwood, 2002). During solar minimum periods, the OMNI data and PFSS model are in better agreement than in periods of higher solar activity.

Figure 4, panels c – e, displays the coronal hole unsigned open flux for the latitude zones demarcated by ${\pm}\,55~\mbox{degrees}$ latitude. Similar evolution patterns are observed with regard to cyclic variation and latitudinal dependence, as compared with measurements of coronal hole areas. In the polar regions, the lack of coverage during the EIT-era dataset is apparent, and is a consequence of the $B$-angle variation. However, the peak values of the EIT coronal hole area and unsigned open flux provide an upper bound on this value for each pole during optimal angling. Considering the upper bounds of the EIT data, good agreement is found with the PFSS open flux in polar regions. This trend continues with AIA/EUVI data, although with more complete coverage of the polar regions. These comparisons allow us to state that the magnetic open flux in polar regions computed with the PFSS model is generally consistent with direct measurements in both solar cycles.

In terms of the solar cycle dependence of open flux in the poles, the unsigned open flux in the northern and southern poles in Solar Cycle 23 evolved in a mostly symmetric manner. An asymmetry presented itself in Solar Cycle 24, however, in the same way as with the evolution of the coronal hole areas. Once again, this is a consequence of asymmetric sunspot emergence. For this cycle, the northern polar unsigned open flux drops off to nearly zero around 2011 and continues at this level. Slight signs of the beginning of a recovery are apparent in 2014, but with no clear recovery trend. The southern pole open flux drops to zero in 2013, two years later than the northern poles, and then begins to rise once again.

Consideration of the low-latitude open flux reveals the discrepancy during periods of maximum solar activity, when low-latitude coronal holes closely border larger concentrations of magnetic flux.

### Signed Magnetic Flux

Figure 5, panels b – d, displays the signed open magnetic flux from each of the latitude zones demarcated by ${\pm}\,55~\mbox{degrees}$. When we consider the northern and southern low-latitude regions of panel c in Figure 5, we see that the signed open flux undergoes a few variations throughout the cycle. During solar minima, the signed open fluxes are relatively weaker because of a reduced low-latitude area during these phases. As the areas increase during Cycle 23, each low-latitude hemisphere begins to exhibit a dominant signed polarity, positive in the south and negative in the north. This behavior decreases during the decline to solar minimum between Cycles 23 and 24. As Cycle 24 begins to increase, observations of low-latitude hemispheric signed flux remain relatively weak.

For the northern and southern polar regions, shown in Figure 5, panels b – d, the polarity dominance is more apparent. During the lull in solar activity around 1997 before Cycle 23 began, each polar region is strongly dominated. The northern pole shows a strong dominance of positive magnetic polarity, while the southern pole shows a strong negative dominance. Our EIT coronal hole and the WSO computed open field measurements agree. As solar maximum for Cycle 23 is reached, this polarity dominance decreases, and each pole becomes neutral around 2000. Throughout this next phase of minimal activity between Cycles 23 and 24, a different picture emerges. Here, the WSO computed open field shows a much weaker polarity dominance, this time of the opposite sign, as expected. This is also reflected in a weaker polar unsigned flux. Our EIT measurements of coronal hole open flux show an even weaker polarity dominance. This stands in stark contrast with the heavy polarity dominance of the previous minimum. As Cycle 24 reaches something resembling a peak around 2014, both poles once again have reverted to a neutral state. Here our AIA/EUVI observations fall more in line with the computed WSO open field measurements.

### A Close Look at Cycle 24

To better illustrate details for Solar Cycle 24, we consider only data from 13 May 2010 – 19 August 2014, that is, Carrington Rotations 2096 – 2154. Here, many of the same trends and conclusions are evident, with some new details visible with scaling. Figures 6, 7, 8, and 9 display identical values to the larger dataset discussed above, but with a retracted viewpoint to only cover the range of data available from AIA/EUVI. Figure 6 shows maps of the latitude distribution of the coronal hole, the mean magnetic field, and the total unsigned flux in the holes. Figures 7, 8, and 9 represent the measured coronal hole area, the unsigned open magnetic flux, and the signed open magnetic flux, respectively. These plots are identical in labeling to those spanning the entire dataset, with the addition of AIA/EUVI raw data displayed in a lighter shade of blue, compared with a 27-day running average in darker blue.

Figure 6, panels b – d, displays the SDO-era data available using our coronal hole detection routine. While our SOHO-era coronal hole data was gathered at a 24-hour cadence, the results were stacked and binned over a full solar rotation. This method allows for the calculation of an upper bound of the coronal hole area and the enclosed unsigned magnetic flux, capturing the location of each Earth-facing coronal hole location throughout that rotation. Our SDO-era method is slightly different, with the inclusion of STEREO/EUVI data. With the inclusion of EUVI data, far-side (opposite of Earth) coronal hole observations are possible. This allows for our coronal hole mappings to be presented at full 12-hour cadence, without binning. This is immediately apparent with the coronal hole area latitude profiles in Figure 6, panel b. In May 2010, the beginning of our dataset, a regular gap in the data appears, coinciding with the solar rotation rate. At the beginning of our SDO-era data, 13 May 2010, the STEREO spacecraft had achieved an orbital separation of roughly 70 degrees ahead and behind, each with respect to Earth. This left a roughly 40-degree range of longitudes that were unobservable in EUV. As the STEREO spacecraft continued in their orbital separation, this gap continued to shrink. With near-limb data truncated from each data source, higher frequency gaps appear further in the dataset as large holes that rotate through these small blind-spots.

SDO-era latitude profiles of coronal hole area and signed/unsigned flux displayed in Figure 6 reveal that a few items are noteworthy. From May 2010 until January 2014, the latitude demarcation of ${\pm}\,55~\mbox{degrees}$ continues to divide our polar and low-latitude coronal holes. From January 2014 until August 2014, two large coronal holes develop at the upper edge of our low-latitude region, then drift and extend beyond ${\pm}\,55~\mbox{degrees}$, with the northern polar extension of this hole developing much weaker and diminishing earlier than the extension found in the southern pole. Referring back to Figure 2, panel b, this same pattern of expansion into the pole is apparent with EIT data from 2001 until 2003. Here, however, this expansion occurs with much greater symmetry and nearly in phase; both the northern and southern extensions into the polar regions evolve and persist. The asymmetric coronal hole distribution within polar regions in Cycle 24 is apparent in the total coronal hole area displayed in Figure 7.

The mean coronal hole field in Figure 6 shows the dominant polarity of each of our major coronal hole distributions. From May 2010 until August 2012, the northern polar coronal hole distribution is dominated by negative magnetic flux, with the southern polar coronal holes dominated by positive flux. Note that the northern polar coronal hole distribution has decreased in area and resulting signed/unsigned flux as of February 2011, with lingering traces thereafter, until it completely vanishes in February 2012. Once again, this particular lead-in to a polar polarity inversion behaves in a much more asymmetric manner than the reversal observed with SOHO-era data in mid-1999.

The unsigned open flux latitude distribution in Figure 6, panel d, results from previous noted trends in the coronal hole area latitude profile. Here we see that the southern polar coronal hole distribution dominates the density of unsigned open magnetic flux, with smaller distributions at the northern pole and lower latitudes. The measured total flux is plotted in Figure 8. As described in Lowder *et al.* (2014), as a whole, the AIA/EUVI measured total flux is much closer to the model computed total flux, but there are variations in the latitude-dependent comparison. The AIA/EUVI measured flux is comparable to or higher than the model computed flux within the polar regions. However, at lower latitudes, the computed flux overshoots the measured flux.

## Conclusions and Discussions

Through the use of SOHO/EIT, SDO/AIA, and STEREO/EUVI A/B EUV data from May 1996 until August 2014, coronal hole boundaries have been tracked over the entirety of Solar Cycle 23 and a good portion of Cycle 24. This extensive range allows for a detailed analysis of coronal hole evolution in all latitudes, and for a comparison of the magnetic open flux, which is directly measured from the coronal holes, from the past cycle to the current cycle. The addition of STEREO/EUVI data allows for improved polar coverage in the current cycle, and also provides a reference to assess the polar hole and open flux measurements by EIT in the past cycle. A few trends in this dataset are apparent.

These data show that coronal holes, and the signed and unsigned magnetic flux measured inside the holes, exhibit distinct evolution patterns as a function of latitude near the polar regions. We also observed an asymmetry in the evolution of coronal holes in the northern and southern polar regions, consistent with observations by McIntosh *et al.* (2014). This asymmetry manifests itself in both the rising of the polar coronal hole coverage during Solar Cycle 23, and more distinctively in the decline and rise of polar coronal holes in Solar Cycle 24. By tracking the evolution of coronal holes across all latitudes, we see the appearance of large dominant-polarity coronal hole structures at lower latitudes, migrating toward the polar regions. While these low-latitude signatures appear at roughly identical times, their push into the polar regions occurs at differing speeds, which causes the northern polar coronal hole to establish itself much earlier.

When the two cycles are compared in their early stages, the current cycle has a similar coronal hole extent and total open flux. Most significantly, in Cycle 24, the polar flux reversal exhibits a stark asymmetry compared with the last cycle. The northern polar holes have dropped off from 2009, two years ahead of the southern polar holes, and the open flux there has remained at a very low level since then, whereas the southern polar holes have recovered from the drop-off in 2013 with the open flux rising again in 2014. It remains to be seen how the differences of Cycle 24 will affect the peak distribution of coronal holes and open magnetic flux during the declining phase of Cycle 24.

Finally, the observed coronal holes, directly measured open flux in these holes, and their latitude dependence have been compared with the calculation by the PFSS model using the synoptic WSO data. We showed that on the one hand, this model reproduces polar coronal holes and open flux in general agreement with observations in both cycles. On the other hand, the clear discrepancy at low latitudes confirms the results in our earlier study (Lowder *et al.*, 2014). This discrepancy significantly contributes to the total open flux, particularly during the solar maximum. The potential field model appears to produce more open flux than observed at the low latitudes in both cycles, even though these regions are presumably better captured by the AIA/EUVI instruments in the current cycle. This comparison therefore indicates the importance of the latitude-dependent evolution of coronal hole boundaries, which provide an observational constraint to help advance models of the Sun’s global magnetic field.

## Figures and Tables

**Figure 10:**
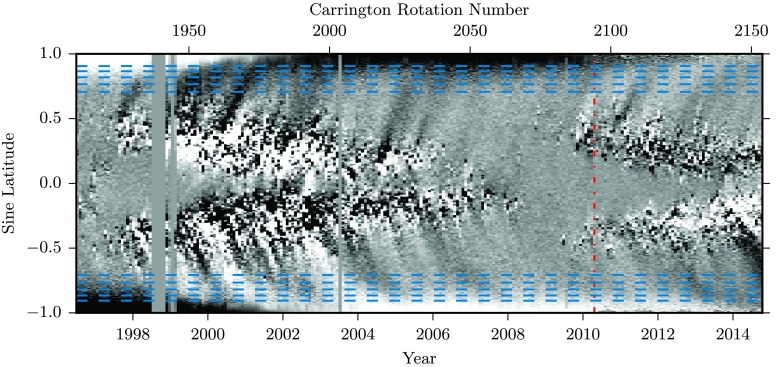
Butterfly diagram (latitude-time profile) of the mean radial magnetic field strength, scaled between ${\pm}\,5~\mbox{G}$. Measurements from the SOHO/MDI transition to those from SDO/HMI at the vertical dot-dashed red line. Latitude boundaries at ${\pm}\,\{45, 50, 55, 60, 65\}~\mbox{degrees}$ are marked by blue dotted lines.

**Figure 11:**
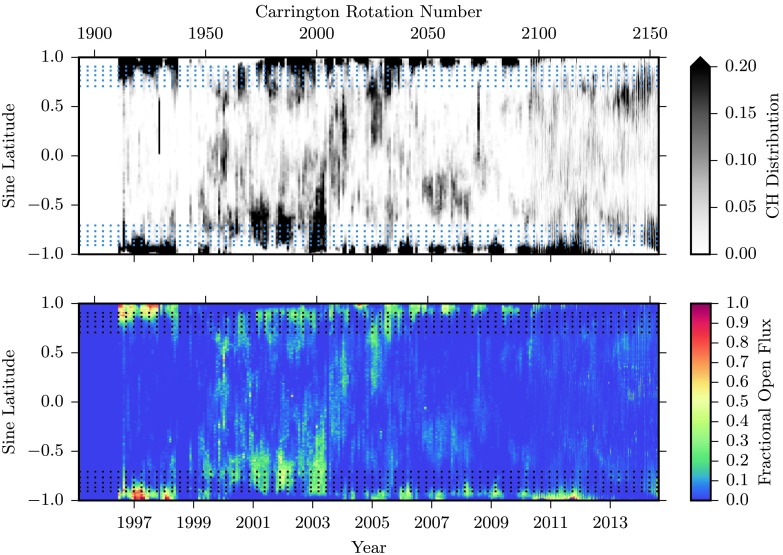
Upper panel: Coronal hole latitude profile of the distribution, integrated over longitude. Lower panel: Latitude profile of coronal hole enclosed magnetic flux, integrated over longitudes. Latitude boundaries of ${\pm}\,\{45, 50, 55, 60, 65\}~\mbox{degrees}$ are marked by blue dashed lines in both panels.

**Figure 12:**
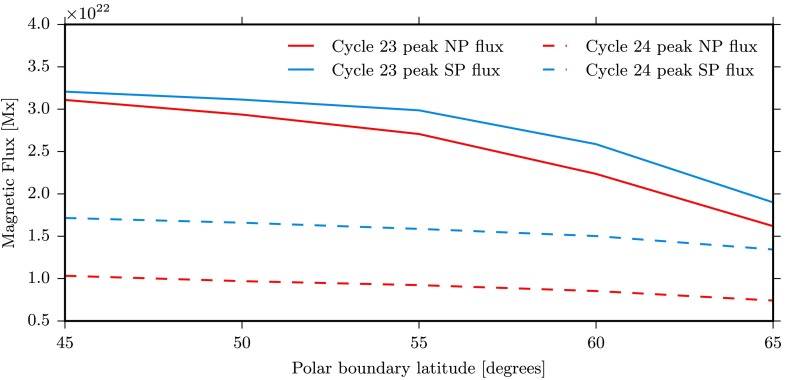
Peak magnitude of the coronal hole enclosed polar magnetic flux for each pole as a function of latitude for the polar boundaries defined from ${\pm}\,\{45, 50, 55, 60, 65\}~\mbox{degrees}$. The northern and southern polar values are mapped in red and blue, respectively. Solar Cycle 23 data are marked with solid lines, while Solar Cycle 24 data are mapped in a dashed fashion. As the polar boundary is pushed further poleward in each direction, this enclosed magnetic flux reduces in magnitude.

## Appendix A: Polar Latitude Determination

In the preceding analysis, a polar boundary latitude was chosen at ${\pm}\,55~\mbox{degrees}$. While the latitude-time profiles display variation over a continuum of latitude bins, a polar boundary value was necessary to quantify the coronal hole area and enclosed flux across specified ranges of latitudes. This appendix works through an analysis of the effects of the shifting polar boundary, providing justification for the choice of boundary.

Figure 10 displays the mean radial magnetic field strength, capped at ${\pm}\,5~\mbox{G}$. Measurements from SOHO/MDI are plotted until 22 April 2010 (marked with a dot-dashed red line), after which SDO/HMI data are mapped. To study a range of latitude values, latitudes of ${\pm}\,\{45, 50, 55, 60, 65\}~\mbox{degrees}$ were chosen as boundaries for study, to give a wide range of values for comparison. These latitude values are marked here by blue dotted lines. During the period from 2001 – 2014, the distribution of strong polar magnetic flux remains above ${\approx}\,60~\mbox{degree}$ latitude. However, a difference in this distribution appears for available data before 2001. Here, the distribution of polar flux appears to dip further equatorward.

This difference in the extent of the polar magnetic field manifests itself in changes of the distribution of the polar open magnetic field. Figure 11 maps out the latitudinal distribution of coronal hole boundaries and the flux enclosed therein throughout the span of observed data. For comparison, latitudes of ${\pm}\,\{45, 50, 55, 60, 65\}~\mbox{degrees}$ are plotted in dotted styles in both panels. Note that the remainder of the data here is identical to the associated panels in Figure 2. From the period 2001 – 2014, the resulting coronal hole distribution remains relatively above ${\pm}\,60~\mbox{degrees}$ latitude. However, in the time spans 1996 – 1998 and 2011 – 2012, extensions of coronal holes dip below this, encroaching closer to ${\pm}\,55~\mbox{degrees}$. The lower panel displays the corresponding coronal hole enclosed unsigned open magnetic flux. These extensions to lower latitudes carry a significant corresponding amount of open magnetic flux.

To quantify the effects of this boundary shift, a profile of open magnetic flux was created for each polar region as defined by the ranges of boundary latitudes previously defined. From each of these profiles, the peak value of the open magnetic flux was separately computed for the range of Solar Cycles 23 and 24. Figure 12 plots these peak values of the polar open magnetic flux as a function of the choice of the polar latitude boundary. The northern and southern polar regions are plotted in red and blue, respectively. Solar Cycle 23 peaks are plotted in solid curves, with Cycle 24 shown by dashed curves. As the polar boundary is pushed further poleward, the peak polar open fluxes decline as more enclosed flux is reclassified as low-latitude flux. For both the northern and southern hemispheres of Solar Cycle 23, a more significant drop in the peak polar open magnetic flux occurs as the polar boundary pushes beyond ${\pm}\,55~\mbox{degree}$ latitude toward the poles. This same trend does not appear for the portion of Cycle 24 data we gathered. For the coronal hole distributions of Cycle 23, moving poleward from ${\pm}\,55~\mbox{degree}$ latitude begins a significant drop in the peak open magnetic flux.

These analyses suggest that a polar boundary of ${\pm}\,55~\mbox{degree}$ latitude provides a balanced choice. It allows for capture of polar open flux during more standard periods of coronal hole activity, while also providing a buffer to capture extensions into slightly lower latitudes without being integrated into lower latitude profiles.
